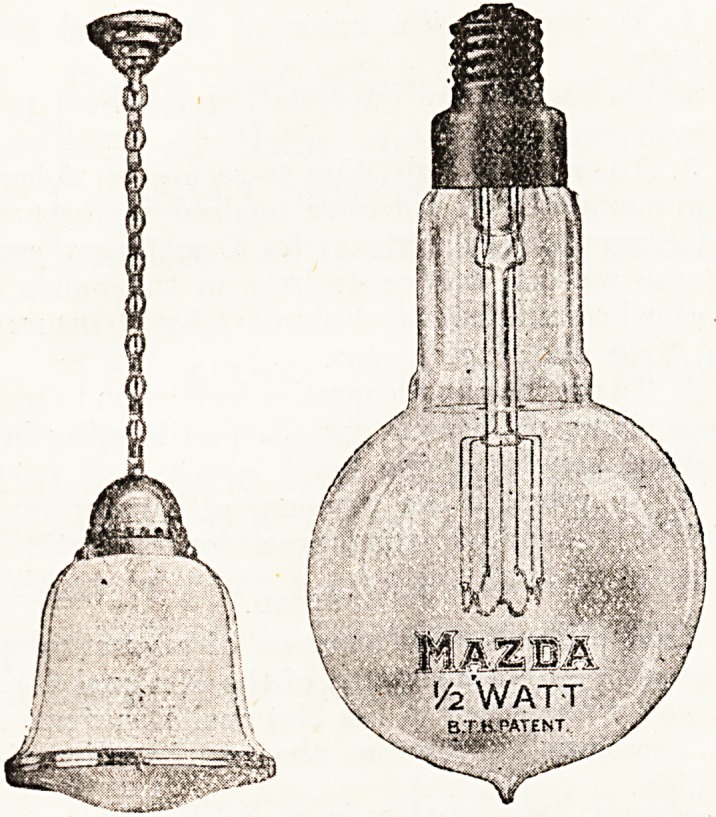# Institutional Needs

**Published:** 1914-03-14

**Authors:** 


### Institutional Needs.

A NEW LAMP FOR HOSPITAL LIGHTING.

A new lamp claiming the same lighting efficiency as the arc lamp and the advantages of the ordinary tungsten lamp is the Mazda "Half-Watt" lamp, which was exhibited by the British Thomson-Houston Co., Ltd., towards the end of last August for the first time in this country at the Company's London offices, Mazda House, 77 Upper Thames Street, E.C. The British Thomson-Houston Company now state that they have standardised the production of these lamps at their Rugby factory, and are in a position to accept orders for them.

The makers state that wherever high-candle powers are required in hospitals and similar institutions, there Mazda "Half-Watt" lamps can be used with advantage. If used instead of arc lamps, the advantage will be in the direction of reduced trouble and maintenance costs; if in place of ordinary tungsten lamps of the same total candle power, then the advantage will take the form of a 50 per cent. reduction in current consumption.

Below are the chief features of the Mazda "Half-Watt" lamp. Efficiency: Half watt per candle or two candles per watt. Average useful life: 800 to 1,000 hours. Quality of light: Owing to the high temperature of the filament, the light is much whiter in colour than that of the ordinary tungsten lamp. The intrinsic brilliancy of the Mazda "Half-Watt" lamp, eight times as high as that of the ordinary tungsten lamp, renders it essential that Mazda "Half-Watt" lamps should be enclosed in suitable diffusing spheres. The characteristic of the Mazda "Half-Watt" lamp is that, instead of consuming between 1 watt and 1.25 watt per candle, it consumes only 0.5. The filament of the Mazda "Half-Watt" lamp is enclosed in a bulb filled with an inert gas such as nitrogen, instead of in a vacuum.

With the placing on the market of the "Half-Watt" lamp, there is now available a range of Mazda lamps in sizes from 1 c.p. to 3,000 c.p., and applicable to every lighting purpose. The Company have also placed on the market a range of special fittings for use with the lamp. The Mazda "Half-Watt" lamp is specially adapted to indirect lighting, and further information, including prices of fittings, will be found in the Company's price list No. 11,000, for copies of which readers are advised to write to Mazda House, 77 Upper Thames Street, E.C.

"BIOMALT."

It has been long recognised that perhaps the most predisposing cause to disease in children and adults is malnutrition, and among the many ingredients which present themselves as a basis for special nutritious diets malt is among the chief. A tonic food in which this is recognised is "Biomalt," which is a compound of maltose, dextrine, organic and inorganic phosphates, lime, ferric oxide, and nitrogenous matter. But the combination of physiologically suitable compounds is not enough; the diet of which they are made must be palatable, a fact of great importance where invalids and children are concerned. "Biomalt" fulfils this condition and has the further advantage that it may be taken not merely by itself, but with cereals or such beverages as coffee, cocoa, or tea. It is obtainable from local chemists, or direct from Regent House, Kingsway, W.C., which is the wholesale depôt, in tins, price 1s. 3d. and 2s. 3d. Neither the prescriber nor heads of institutional staffs can afford to overlook this preparation, which provides a tonic for the overworked, as well as a food for the anæmic.

## Figures and Tables

**Figure f1:**